# Supplementary material for: Boosting spatial and energy resolution in STM with a double-functionalized probe
Source: Sci Adv. 2024 Aug 28;10(35):eadq6975. doi: 10.1126/sciadv.adq6975 (PMC11352829; doi:10.1126/sciadv.adq6975)
Supplement: Supplementary file 1 — Supplementary Text Figs. S1 to S6 [file sciadv.adq6975_sm.pdf]

Science Advances
AAAS

Supplementary Materials for

Boosting spatial and energy resolution in STM with a double-functionalized probe

Artem Odobesko *et al.*

Corresponding author: Artem Odobesko, artem.odobesko@uni-wuerzburg.de

Sci. Adv. **10**, eadq6975 (2024)
DOI: 10.1126/sciadv.adq6975

This PDF file includes:

Supplementary Text
Figs. S1 to S6

— Supplementary Material —

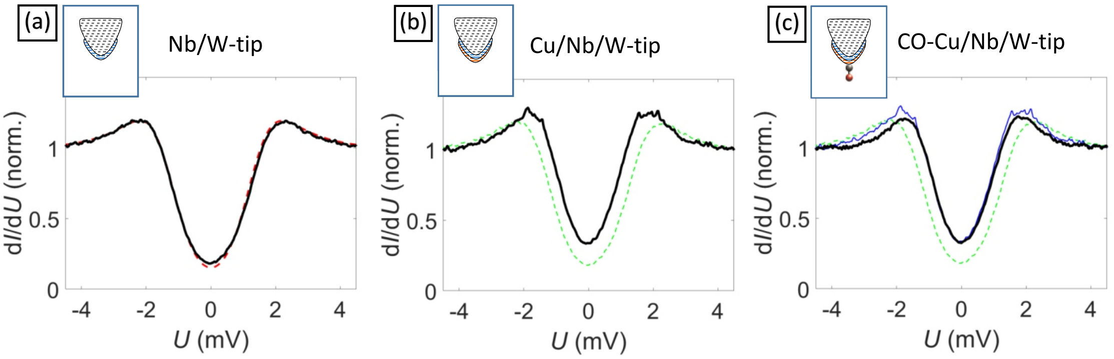

FIG. S1. **Superconducting properties of functionalized probes.** Tunneling spectra (black solid lines) of the different functionalized probes measured atop the clean Cu(001) surface at $T = 4.2\,\mathrm{K}$. (a) Tungsten tip functionalized with a superconducting Nb cluster. The red dashed line indicates the BCS fitting curve. (b) Same tip as in (a) after gentle poking into the Cu(001) surface, green dashed curve corresponds to Nb/W-probe before poking into Cu surface. (c) Same tip as in (b) with CO molecule attached to it. Blue curve corresponds to Cu/Nb/W-probe before attaching CO. Fitting and setting parameters: (a) $\Delta_{\mathrm{tip}} = 1.4\,\mathrm{meV}$, $\Gamma_{\mathrm{Dynes}} = 1\,\mathrm{meV}$, $T_{\mathrm{eff}} = 4.7\,\mathrm{K}$; (a-c) $I_{\mathrm{set}} = 0.4\,\mathrm{nA}$, $U_{\mathrm{set}} = -7\,\mathrm{mV}$, $U_{\mathrm{mod}} = -0.1\,\mathrm{mV}$.

A superconducting Nb probe is prepared on the Nb(110) surface by intending a tungsten tip into the Nb surface by a depth of about $(1-2)\,\mu\mathrm{m}$, thereby creating a large Nb cluster at the tip apex. The measured tip gap Δ_{tip} of the SC functionalized probes [black solid curve in Fig. S1(a)] can be reasonably well fitted by a BCS model with a quasiparticle broadening encoded in the Dynes parameter Γ_{Dynes} and a thermal broadening $k_{\mathrm{B}}T_{\mathrm{eff}}$ [red dashed curve in Fig. S1(a)]. Picking up a CO molecule is carried out on the Cu(001) surface, since on a Nb(110) surface this procedure was unsuccessful. To pick-up CO with a Nb/W-tip, we first gently poke the tip into the Cu(001) surface, in order to cover the Nb apex with a thin Cu layer. This causes a small reduction of the tip SC gap value down to $\Delta_{\mathrm{tip}} = 1.2\,\mathrm{meV}$ [black curve in Fig. S1(b)]. With such a Cu/Nb/W-tip we are able to pick up a CO molecule. We use the following procedure: The tip is stabilized at $U = -2\,\mathrm{V}$ and $I = 1\,\mathrm{nA}$ above the molecule, then the feedback is switched off and the bias voltage is increased to $-4\,\mathrm{V}$. The successful transfer of the CO molecule to the tip apex is indicated by a sudden jump of the tunneling current to a lower absolute value. The attached CO molecule to the Cu/Nb/W-

tip does not significantly change the shape of the SC gap of such a probe [black curve in Fig. S1(c)] compared to the same probe without CO [blue curve in Fig. S1(c)].

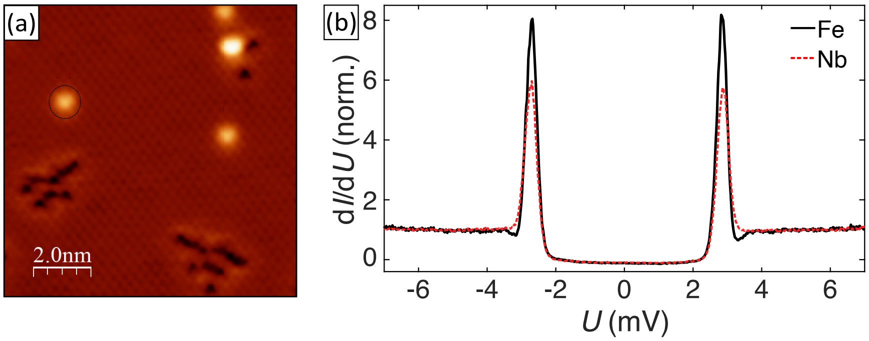

FIG. S2. **Spectrum of single Fe atom.** (a) STM topographic image of singe Fe atoms on the atomically clean Nb(110) surface. (b) STS spectra measured with SC-probe on top of Fe atom (black line) indicated with circle in (a) and on clean Nb far from any defect (red dotted line).

The weak hybridization of an Fe atom with the Nb substrate gives rise to a pair of YSR bound states positioned alongside the coherence peaks of superconducting Nb, as examined closely in Refs.[9, 33, 34]. In Fig.S2(b) the dI/dU signal atop of the Fe atom (black line) is enhanced as compared to the spectrum of the clean Nb (red dashed line) at the position of coherence peaks at located at $\Delta_{\mathrm{tip}+\Delta_{\mathrm{Nb}}}$ energy. These YSR resonances originate mostly from the Fe atom's d_{z^2} orbital when Fe atom is adsorbed in the energetically preferred the four-fold hollow adsorption site within the Nb(110) unit cell.

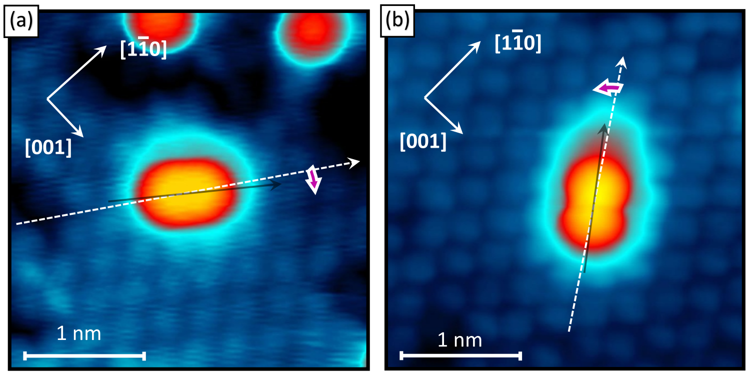

FIG. S3. **High-resolution topography of Fe dimers** STM topographic images of two Fe dimers 'A' and 'B' oriented along the [1$\bar{1}$1] and the [1$\bar{1}\bar{1}$] directions, respectively. (a) The axis of dimer 'A' (marked by a dark arrow) is rotated clockwise by about 4° from [1$\bar{1}$1](marked by a white dashed arrow). (b) For the type 'B' dimer the rotation is counterclockwise from [1$\bar{1}\bar{1}$] by the same value.

The relative inward displacement of a pair of Fe atoms from the four-fold hollow adsorption site leads to a small deviation of the dimer axis (black solid arrows Fig. S3) by $\approx 4°$ from the given directions [1$\bar{1}$1] and [1$\bar{1}\bar{1}$], as marked by whie dashed arrows in Fig. S3. This is generally observed for all close-pack Fe dimers found on the surface.

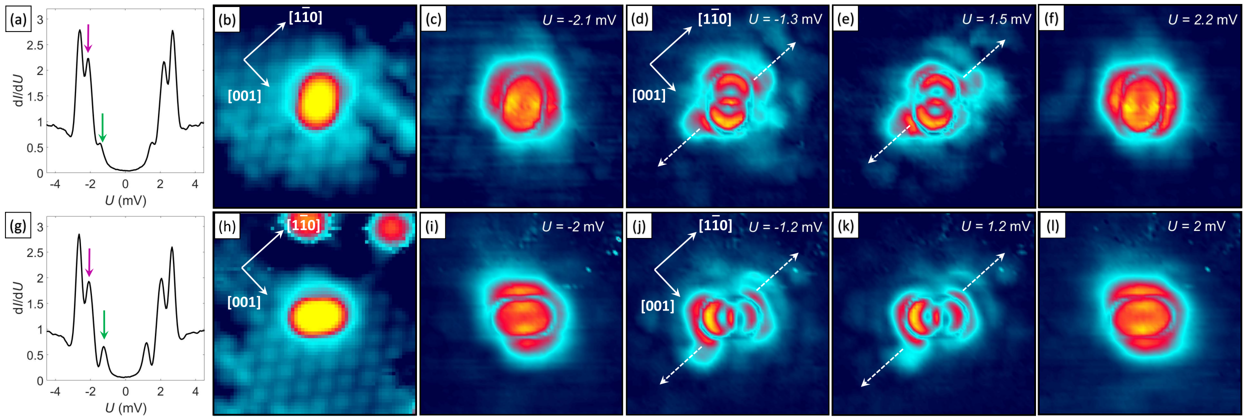

FIG. S4. **DOS maps of YSR states of Fe dimers.** Point tunneling spectra (a,g), STM topography images (b,h) and spatial dI/dU maps (c-f and i-l) of two mirror symmetric Fe dimers on a clean Nb(110) surface oriented in [1$\bar{1}\bar{1}$] direction (upper row) and along [1$\bar{1}$1] (bottom row). Setpoint parameters: $U_{\mathrm{set}} = 7\,\mathrm{mV}$, $I_{\mathrm{set}} = 1\,\mathrm{nA}$.

The data in Fig. S4 represent spatial maps of hole- and electron-like YSR resonances measured at negative and positive tunneling energies. It is evident that the spatial patterns of the dI/dU maps for both hole-like and electron-like YSR resonances are identical. Additionally, the "focusing effect," characterized by less attenuation of the oscillating YSR wave function in a specific direction (marked by dashed arrows in Fig. S4(d-e, j-k)), is clearly observed for low-energy YSR resonances. This effect occurs in the same direction along [1$\bar{1}$0], regardless of the dimer orientation.

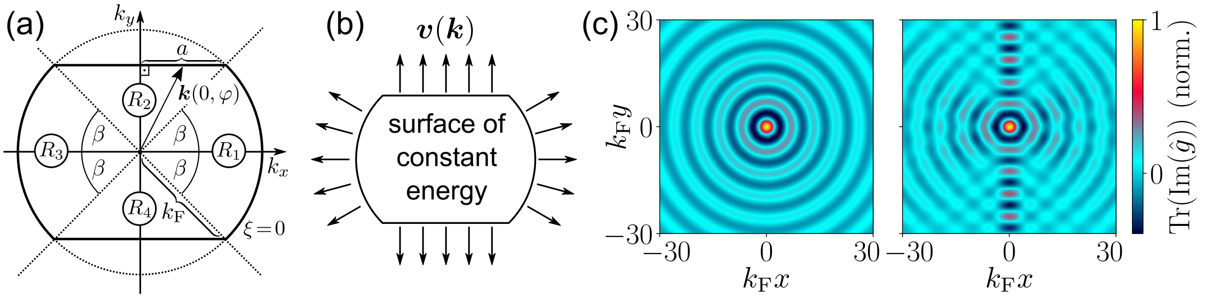

FIG. S5. **Model of anisotropic Fermi surface** (a) Stadium-shape Fermi surface at the energy $\xi = 0$ with two circular sectors $R_{1,3}$ and two flat segments $R_{2,4}$. $a \in [0, k_{\mathrm{F}}]$ and the angle $\beta = \arccos(a/k_{\mathrm{F}})$ define the shape of the stadium, where $a = 0$ corresponds to a complete circular Fermi surface with radius $k_{\mathrm{F}} > 0$ and $\beta = \pi/2$. The Fermi surface is parametrized by $\boldsymbol{k}(\xi, \varphi)$ in Eq. (S5). (b) The direction of the group velocity $\boldsymbol{v}(\boldsymbol{k})$ is always perpendicular to the surface of constant energy, see Eq. (S4). (c) Bare Green's function in Eq. (S10) for $a = 0\,k_{\mathrm{F}}$ (left) and $a = 0.6\,k_{\mathrm{F}}$ (right). Common parameters: $E = 0\,\Delta$, $\eta = 10^{-3}\Delta$, $\xi_0 = 100/k_{\mathrm{F}}$.

Bare substrate Green's function

We describe the superconducting Nb surface by a continuous 2D model with quasimomentum space $\boldsymbol{k} = (k_x, k_y) \in \mathbb{R}^2$ and real space $\boldsymbol{r} = (x, y) \in \mathbb{R}^2$. Given a dispersion relation $\xi(\boldsymbol{k})$, the position-dependent Green's function of a homogeneous two-dimensional superconductor follows from an inverse Fourier transformation

$$\hat{g}(\boldsymbol{r}, z) = \frac{1}{(2\pi)^2} \int_{\mathbb{R}^2} d^2k\, e^{i\boldsymbol{k}\cdot\boldsymbol{r}} \hat{g}(\xi(\boldsymbol{k}), z), \qquad \hat{g}(\xi, z) = \frac{-\sigma_0 \otimes [z\tau_0 + \xi\tau_3 + \Delta\tau_1]}{\xi^2 + \Delta^2 - z^2}, \tag{S1}$$

where $\hat{g}(\xi, z)$ is the Gor'kov Green's function of the bare superconductor [56]. Furthermore, σ_j and τ_j ($j = 1, 2, 3$) are Pauli matrices in spin and Nambu space with the corresponding identities σ_0 and τ_0. All matrices are defined in the basis $(\psi_\uparrow, \psi_\downarrow^\dagger, \psi_\downarrow, -\psi_\uparrow^\dagger)$, where $\psi_\sigma^{(\dagger)}$ is the annihilation (creation) field operator of an electron with spin σ. Furthermore, $\Delta > 0$ is the superconducting pairing and $z = E + i\eta$, with energy $E \in \mathbb{R}$ and Dynes parameter $\eta \to 0^\pm$ [57] that defines the retarded ($\eta > 0$) and advanced ($\eta < 0$) Green's function. In addition, we define the BCS Green's function

$$\hat{g}_{\mathrm{BCS}}(z) = \frac{1}{\pi} \int_{\mathbb{R}} d\xi\, \hat{g}(\xi, z) = \frac{-\sigma_0 \otimes (z\tau_0 + \Delta\tau_1)}{\sqrt{\Delta^2 - z^2}}. \tag{S2}$$

To obtain a parametrization of the 2D integral in Eq. (S1), we define the surface of constant energy at the Fermi energy by dividing it into four regions R_j as depicted in

Fig. S5(a), i.e., $\mathbb{R}^2 = R_1 \cup R_2 \cup R_3 \cup R_4$. The energy dispersion in these regions is assumed to take the form

$$\xi(\boldsymbol{k}) = \begin{cases} \frac{\hbar^2}{2m}(k_x^2 + k_y^2) - E_\mathrm{F} & , \ \boldsymbol{k} \in R_1 \cup R_3 \\ \frac{\hbar^2}{2m} k_y^2 - E_\mathrm{F} & , \ \boldsymbol{k} \in R_2 \cup R_4 \end{cases} , \tag{S3}$$

where $E_\mathrm{F} = \hbar^2 k_\mathrm{F}^2/(2m)$ is the Fermi energy at the Fermi quasimomentum $k_\mathrm{F} > 0$ of a circular Fermi surface, $\hbar$ is the Planck constant, and m is the effective electron mass. As sketched in Fig. S5(b), a finite $a > 0$ will result in a preferred wave propagation along the y-direction (focusing effect) according to the group velocity

$$\boldsymbol{v}(\boldsymbol{k}) = \frac{1}{\hbar}\nabla_{\boldsymbol{k}}\xi(\boldsymbol{k}) = \begin{cases} \frac{\hbar \boldsymbol{k}}{m} & , \ \boldsymbol{k} \in R_1 \cup R_3 \\ \frac{\hbar k_y}{m}\hat{\boldsymbol{e}}_y & , \ \boldsymbol{k} \in R_2 \cup R_4 \end{cases} . \tag{S4}$$

We parametrize all regions in polar-like coordinates $\boldsymbol{k}(\xi, \varphi) = k(\xi, \varphi)(\cos(\varphi), \sin(\varphi))$ with the polar angle $\varphi \in [-\pi, \pi)$. Substituting the parametrization into the dispersion relation and solving for the angle-dependent magnitude $k(\xi, \varphi)$, we get

$$\boldsymbol{k}(\xi, \varphi) = \begin{cases} k_\mathrm{F}\sqrt{1 + \xi/E_\mathrm{F}} \begin{pmatrix} \cos(\varphi) \\ \sin(\varphi) \end{pmatrix} & , \ \boldsymbol{k} \in R_1 \cup R_3 \\ \frac{k_\mathrm{F}}{|\sin(\varphi)|}\sqrt{1 + \xi/E_\mathrm{F}} \begin{pmatrix} \cos(\varphi) \\ \sin(\varphi) \end{pmatrix} & , \ \boldsymbol{k} \in R_2 \cup R_4 \end{cases} . \tag{S5}$$

Using this parametrization, the bare Green's function in Eq. (S1) becomes

$$\hat{g}(\boldsymbol{r}, z) = \frac{m}{h^2}\sum_{n=1}^{2}\int_{\Phi_n} d\varphi\, A_n(\varphi)\int_{-E_F}^{\infty} d\xi\, \hat{g}(\xi, z)\,(e^{i\boldsymbol{r}\cdot\boldsymbol{k}(\xi,\varphi)} + e^{-i\boldsymbol{r}\cdot\boldsymbol{k}(\xi,\varphi)}), \tag{S6}$$

with the angle intervals $\Phi_1 = [-\beta, \beta)$, $\Phi_2 = [\beta, \pi - \beta)$ and the Jacobians $A_1(\varphi) = 1$, $A_2(\varphi) = 1/\sin^2(\varphi)$, respectively. The angle $\beta = \arccos(a/k_\mathrm{F})$ is defined by the stadium parameter $a \in [0, k_\mathrm{F}]$ of the flat segments, as sketched in Fig. S5(a). We approximate $\sqrt{1 + \xi/E_\mathrm{F}} \approx 1 + \xi/(2E_\mathrm{F})$ by assuming that $E_\mathrm{F} \to \infty$ is the largest energy scale (Andreev approximation). All energy integrals over ξ are then of the form

$$\int_{\mathbb{R}} d\xi\, \hat{g}(\xi, z) e^{\pm i\boldsymbol{k}_n\cdot\boldsymbol{r}} \overset{\text{(S1)}}{=} -\, e^{\pm i k_F b_n}\sigma_0 \otimes \int_{\mathbb{R}} d\xi\, \frac{z\tau_0 + \Delta\tau_1 + \xi\tau_3}{\xi^2 + u^2}\, e^{\pm i\kappa_n\xi}, \tag{S7}$$

where $u = \sqrt{\Delta^2 - z^2}$, $\kappa_n = k_\mathrm{F} b_n/(2E_\mathrm{F})$, $b_1 = x\cos(\varphi) + y\sin(\varphi)$, and $b_2 = x\cot(\varphi) + y$. The two different integrals are solved using complex contour integration, resulting in

$$\int_{\mathbb{R}} d\xi\, \frac{e^{\pm i\kappa_n\xi}}{\xi^2 + u^2} = \frac{\pi}{u}e^{-|\kappa_n|u}, \qquad \int_{\mathbb{R}} d\xi\, \frac{\xi\, e^{\pm i\kappa_n\xi}}{\xi^2 + u^2} = \pm i\pi\,\mathrm{sgn}(\kappa_n)\, e^{-|\kappa_n|u}. \tag{S8}$$

In summary, using $\mathrm{sgn}(\kappa_n) = \mathrm{sgn}(b_n)$ since $k_\mathrm{F} > 0$ and $E_\mathrm{F} > 0$, we get

$$\int_{\mathbb{R}} d\xi\, \hat{g}(\xi, z) e^{\pm i \boldsymbol{k}_n \cdot \boldsymbol{r}} \overset{\text{(S2)}}{=} -\pi\, e^{\pm i k_\mathrm{F} b_n} \Big(-\hat{g}_\mathrm{BCS}(z) \pm i (\sigma_0 \otimes \tau_3)\, \mathrm{sgn}(b_n) \Big) e^{-k_\mathrm{S}(z)\, |b_n|}, \tag{S9}$$

where $k_\mathrm{S}(z) = \sqrt{\Delta^2 - z^2}/(\hbar v_\mathrm{F})$ is the inverse coherence length of the superconductor and $v_\mathrm{F} = 2E_\mathrm{F}/(\hbar k_\mathrm{F})$ is the Fermi velocity for a circular Fermi contour ($a = 0$). Note that $\xi_0 := 1/k_\mathrm{S}(0) = \hbar v_\mathrm{F}/\Delta$ is the corresponding coherence length at the Fermi energy. In total, the bare Green's function of the superconducting substrate reads

$$\hat{g}(\boldsymbol{r}, z) = N_0 \sum_{n=1}^{2} \int_{R_n} d\varphi\, A_n(\varphi) \Big(\hat{g}_\mathrm{BCS}(z) \cos(k_\mathrm{F}|b_n|) + (\sigma_0 \otimes \tau_3) \sin(k_\mathrm{F}|b_n|) \Big)\, e^{-k_\mathrm{S}(z)\, |b_n|}, \tag{S10}$$

where we defined $N_0 = 2\pi m/h^2$, which is the normal-state density of states per unit area and spin at the Fermi energy. The angle integral over φ is then solved numerically. The spatial behavior of the bare Green's function for different stadium parameters a is shown in Fig. S5(c) and we observe the focusing effect along the y-direction for $a > 0$.

Energy-dependent phase shift between electrons and holes

In general, there is an energy-dependent phase shift between the rapid oscillating electron and hole components of the bare substrate Green's function, which was experimentally observed in the presence of YSR impurities [17]. These oscillations are described by the integrand in Eq. (S10), which reads

$$\hat{g}_\mathrm{BCS}(z) \cos(k_\mathrm{F}|b_n|) + (\sigma_0 \otimes \tau_3) \sin(k_\mathrm{F}|b_n|) \overset{\text{(S2)}}{=} \sigma_0 \otimes \begin{pmatrix} \frac{-z \cos(k_\mathrm{F}|b_n|)}{\sqrt{\Delta^2 - z^2}} + \sin(k_\mathrm{F}|b_n|) & \frac{-\Delta \cos(k_\mathrm{F}|b_n|)}{\sqrt{\Delta^2 - z^2}} \\ \frac{-\Delta \cos(k_\mathrm{F}|b_n|)}{\sqrt{\Delta^2 - z^2}} & \frac{-z \cos(k_\mathrm{F}|b_n|)}{\sqrt{\Delta^2 - z^2}} - \sin(k_\mathrm{F}|b_n|) \end{pmatrix}. \tag{S11}$$

Recall that $z = E + i\eta$ with $\eta \to 0^\pm$. At this stage, we already observe that the diagonal components at small energies $E \approx 0$ oscillate $\propto \sin(k_\mathrm{F}|b_n|)$, while at energies close to the gap $E \approx \pm\Delta$, the oscillations $\propto \cos(k_\mathrm{F}|b_n|)$ dominate. This becomes more obvious if we rewrite the diagonal elements as

$$\frac{-z}{\sqrt{\Delta^2 - z^2}} \cos(k_\mathrm{F}|b_n|) \pm \sin(k_\mathrm{F}|b_n|) = \sqrt{1 + \sinh^2(\gamma)}\, \cos(k_\mathrm{F}|b_n| \mp \alpha), \tag{S12}$$

with the energy-dependent amplitude defined via $\sinh(\gamma) = -z/\sqrt{\Delta^2 - z^2}$ and the energy-dependent phase shift defined by $\cos(\alpha) = \sinh(\gamma)/\sqrt{\sinh^2(\gamma) + 1}$. The relative phase shift between electrons and holes is 2α. Note that it is maximal at zero energy $E = 0$, for which $\alpha = \pi/2$, hence, $2\alpha = \pi$. For $E = \pm\Delta$, we get $\alpha = 0, \pi$, such that $2\alpha = 0, 2\pi$ and there is no relative phase shift.

Finally, for large energies away from the superconducting gap, $|E| \to \infty$ and $-z/\sqrt{\Delta^2 - z^2} \to -i\,\mathrm{sgn}(\eta)$, we obtain the expected normal-metal result

$$\hat{g}_{\mathrm{BCS}}(z)\cos(k_{\mathrm{F}}|b_n|) + (\sigma_0 \otimes \tau_3)\sin(k_{\mathrm{F}}|b_n|) \to -i\,\mathrm{sgn}(\eta)\,\sigma_0 \otimes e^{i\,\mathrm{sgn}(\eta)k_{\mathrm{F}}|b_n|\tau_3}, \tag{S13}$$

which shows that electrons and holes are moving in opposite directions.

Dressed substrate Green's function with magnetic impurities

Following Ref. [21], we add $N \in \mathbb{N}_0$ magnetic impurities at positions $\boldsymbol{r}_j$ on the bare substrate via the self-energy

$$\hat{\Sigma}(\boldsymbol{r}) = \sum_{j=1}^{N} \hat{\Sigma}_j\,\delta(\boldsymbol{r} - \boldsymbol{r}_j), \qquad \hat{\Sigma}_j = \boldsymbol{J}_j \cdot (\boldsymbol{\sigma} \otimes \tau_0) + U_j(\sigma_0 \otimes \tau_3). \tag{S14}$$

Here, the parameters $\boldsymbol{J}_j = (J_{1,j}, J_{2,j}, J_{3,j})$ and U_j are the magnetic exchange field and effective onsite potential, respectively, of the j-th impurity. Then, the dressed substrate Green's function $\hat{G}(\boldsymbol{r}, \boldsymbol{r}', z)$ is determined from the Dyson equation as

$$\begin{aligned}\hat{G}(\boldsymbol{r}, \boldsymbol{r}', z) &= \hat{g}(\boldsymbol{r} - \boldsymbol{r}', z) + \int_{\mathbb{R}^2} d^2r''\,\hat{g}(\boldsymbol{r} - \boldsymbol{r}'', z)\hat{\Sigma}(\boldsymbol{r}'')\hat{G}(\boldsymbol{r}'', \boldsymbol{r}', z) \\ &= \hat{g}(\boldsymbol{r} - \boldsymbol{r}', z) + \sum_{j=1}^{N} \hat{g}(\boldsymbol{r} - \boldsymbol{r}_j, z)\hat{\Sigma}_j\hat{G}(\boldsymbol{r}_j, \boldsymbol{r}', z).\end{aligned} \tag{S15}$$

Evaluating this expression at all impurity positions $\boldsymbol{r} = \boldsymbol{r}_j$, we obtain the closed system of equations that is rearranged into the matrix equation

$$\begin{pmatrix} \hat{1} - \hat{g}(0,z)\hat{\Sigma}_1 & -\hat{g}(\boldsymbol{r}_1 - \boldsymbol{r}_2, z)\hat{\Sigma}_2 & \cdots & -\hat{g}(\boldsymbol{r}_1 - \boldsymbol{r}_N, z)\hat{\Sigma}_N \\ -\hat{g}(\boldsymbol{r}_2 - \boldsymbol{r}_1, z)\hat{\Sigma}_1 & \hat{1} - \hat{g}(0,z)\hat{\Sigma}_2 & \cdots & -\hat{g}(\boldsymbol{r}_2 - \boldsymbol{r}_N, z)\hat{\Sigma}_N \\ \vdots & \vdots & \ddots & \vdots \\ -\hat{g}(\boldsymbol{r}_N - \boldsymbol{r}_1, z)\hat{\Sigma}_1 & -\hat{g}(\boldsymbol{r}_N - \boldsymbol{r}_2, z)\hat{\Sigma}_2 & \cdots & \hat{1} - \hat{g}(0,z)\hat{\Sigma}_N \end{pmatrix} \begin{pmatrix} \hat{G}(\boldsymbol{r}_1, \boldsymbol{r}', z) \\ \hat{G}(\boldsymbol{r}_2, \boldsymbol{r}', z) \\ \vdots \\ \hat{G}(\boldsymbol{r}_N, \boldsymbol{r}', z) \end{pmatrix} = \begin{pmatrix} \hat{g}(\boldsymbol{r}_1 - \boldsymbol{r}', z) \\ \hat{g}(\boldsymbol{r}_2 - \boldsymbol{r}', z) \\ \vdots \\ \hat{g}(\boldsymbol{r}_N - \boldsymbol{r}', z) \end{pmatrix}. \tag{S16}$$

Solving this system for all $\hat{G}(\boldsymbol{r}_j, \boldsymbol{r}', z)$ and substituting the solution into Eq. (S15) provides the full solution of the dressed surface Green's function $\hat{G}(\boldsymbol{r}, \boldsymbol{r}', z)$ including N impurities. For $\eta > 0$, we define the retarded and advanced Green's functions as $\hat{G}^{r,a}(\boldsymbol{r}, \boldsymbol{r}', E) = \hat{G}(\boldsymbol{r}, \boldsymbol{r}', E \pm i\eta)$, with which we define the spectral function

$$\hat{\rho}(\boldsymbol{r}, \boldsymbol{r}', E) = \frac{1}{2\pi i}\Big[\hat{G}^a(\boldsymbol{r}, \boldsymbol{r}', E) - \hat{G}^r(\boldsymbol{r}, \boldsymbol{r}', E)\Big]. \tag{S17}$$

The total electronic local density of states (LDOS) $\rho(\boldsymbol{r}, E) = \rho_\uparrow(\boldsymbol{r}, E) + \rho_\downarrow(\boldsymbol{r}, E)$ for electrons of spin $\sigma \in \{\uparrow, \downarrow\}$ at position $\boldsymbol{r}$ readily follows from the corresponding elements of the spectral function $\hat{\rho}(\boldsymbol{r}, \boldsymbol{r}', E)$ evaluated at $\boldsymbol{r} = \boldsymbol{r}'$. In particular, it holds that

$$\rho_\sigma(\boldsymbol{r}, E) = \lim_{\eta \to 0^+} \hat{\rho}_{\sigma\sigma,\mathrm{ee}}(\boldsymbol{r}, \boldsymbol{r}, E) = \frac{1}{\pi} \lim_{\eta \to 0^+} \mathrm{Im}\big[\hat{G}^a_{\sigma\sigma,\mathrm{ee}}(\boldsymbol{r}, \boldsymbol{r}, E)\big]. \tag{S18}$$

In the following, we focus on the case of $N = 2$ identical impurities with the same exchange coupling $\boldsymbol{J}_j = J\boldsymbol{e}_3$ (along the z-direction) and the same onsite potential $U_j = U$, which are a distance d apart from each other.

In general, the two individual YSR impurity states hybridize and form four subgap states at energies $|E_n| < \Delta$ with wave functions ψ_n with $n \in \{1, 2, 3, 4\}$, which appear as peaks in the LDOS in Fig. S6(c) [cf. Fig. 3(b) in the main text]. Furthermore, the wave functions ψ_n are obtained from the LDOS when evaluated at the bound state energy E_n, i.e., $\rho(\boldsymbol{r}, E_n) \propto |\psi_n|^2$. For the later discussion of the orbitals of the tip wave function we also need their first derivatives $\partial_\alpha \psi$ for $\alpha \in \{x, y\}$, which are obtained from

$$\left|\frac{\partial \psi_n(\boldsymbol{r})}{\partial \alpha}\right|^2 \propto \sum_\sigma \frac{\partial}{\partial \alpha}\frac{\partial}{\partial \alpha'} \hat{\rho}_{\sigma\sigma,\mathrm{ee}}(\boldsymbol{r}, \boldsymbol{r}', E_n)\Big|_{\boldsymbol{r}=\boldsymbol{r}'}. \tag{S19}$$

In Fig. S6(a) we show both $|\psi_n|^2$ (upper row) and the gradient of the wave functions $|\nabla\psi_n|^2 = |\partial_x \psi_n|^2 + |\partial_y \psi_n|^2$ (lower row) of all four bound states. There are two YSR states with even spatial symmetry and two YSR states with odd spatial symmetry. Furthermore, we also observe the aforementioned focusing effect as well as the phase shift between states with positive and negative energy. Note that the LDOS in Fig. S6(c) does not show the typical superconductor coherence peaks at $E = \pm\Delta$ in the presence of subgap states due to the conservation of the total number of states.

Our goal is to estimate the Fermi quasimomentum k_F of the substrate from the experimental tunneling conductance spectra shown in Fig. S3 and Fig. 2 in the main text. For the chosen set of parameters indicated in Fig. S6, we find a reasonably good agreement between

the theoretical spatial LDOS at negative energies [1 and 2 in Fig. S6(a)] and experimental conductance spectra at negative voltage bias [Figs. S2(g) and 2(h) in the main text]. In particular, matching the theoretical ($d = 2.5/k_\mathrm{F}$) and experimental ($d \approx 2.48$-$2.85\,\mathrm{Å}$) data allows us to estimate $k_\mathrm{F} \approx 9.4\,\mathrm{nm}^{-1}$.

The experimental data at positive voltage bias (Fig. S4) shows no qualitative differences to their counterpart at bias (Fig. 2 in the main text). In particular, we do not observe the expected phase shift in our measurement that should be visible based on our calculations presented in Fig. S6.

Fig. S6(b) shows $|\psi_n|^2$ (red lines) and $|\nabla\psi_n|^2$ (blue lines) for all four hybridized YSR subgap states along the dimer axis for odd states and along the nodal axis for even states. Those axes are depicted as white arrows in Fig. S6(a). To compare them with the experimental $\mathrm{d}I/\mathrm{d}U$ measurements along these axes (black lines), we use the estimated central value of $k_\mathrm{F} = 9.4\,\mathrm{nm}^{-1}$ to scale the theory curves to SI units. From a direct comparison with the theoretical data, we see that the experimental data does not show any constant periodicity along the measurement lines. This effect could be caused by the magnetic dimer locally modifying the Fermi surface. Therefore, moving closer or away from the dimer leads to a modification of the substrate Fermi wavenumber k_F. We also see that the oscillatory pattern suggests that also transport through p-orbitals contributes to the full signal, as discussed below.

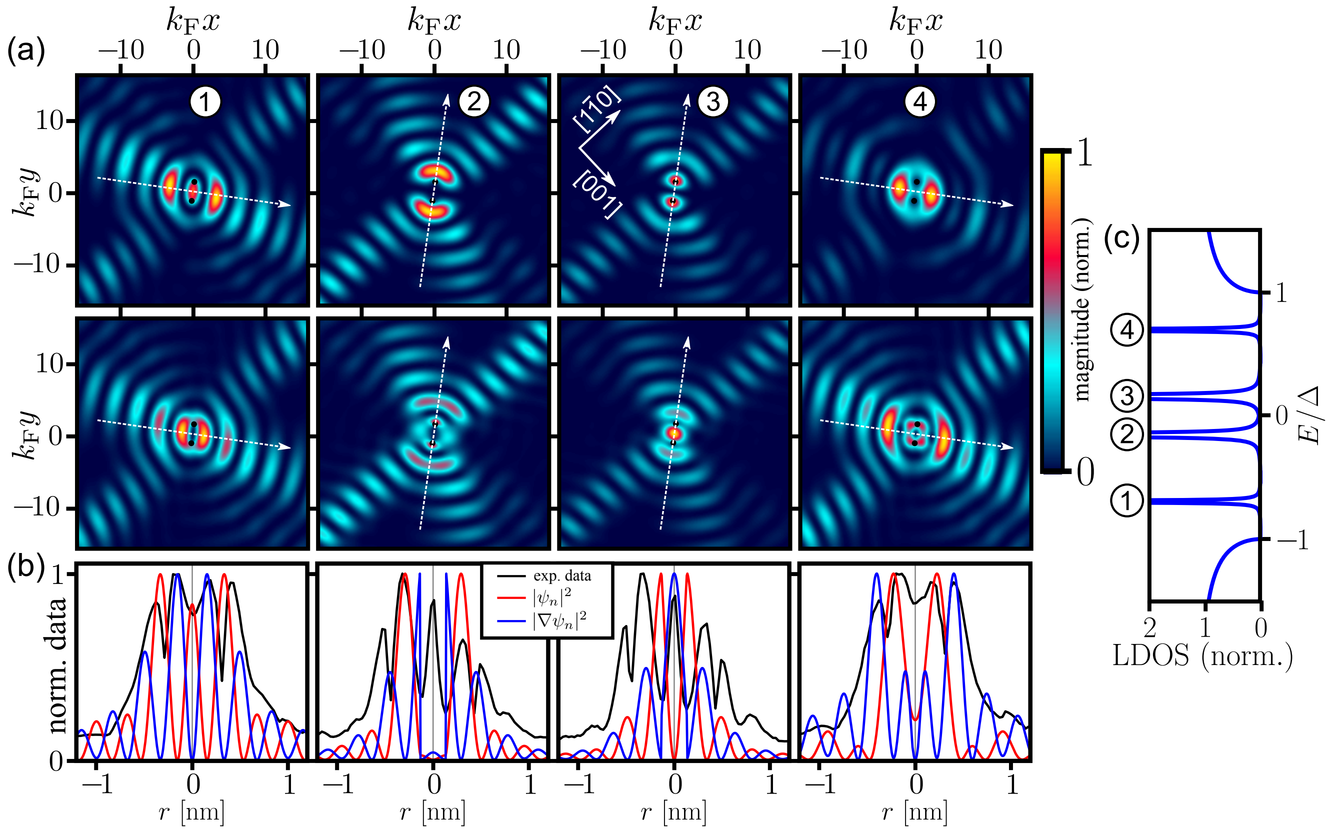

FIG. S6. **Calculated spatial maps of YSR wave function and its gradient.** (a) Wave function $|\psi_n|^2$ (upper row) and its gradient $|\nabla\psi_n|^2$ (lower row) at the four YSR energies [indicated by 1,2,3,4 in panel (c)] from negative to positive (see main text, Fig. 5b). Black dots represent the locations $\boldsymbol{r}_{1,2}$ of the magnetic impurities. (b) Measured $\mathrm{d}I/\mathrm{d}U$ (black) and theoretical $|\psi_n|^2$ (red) and $|\nabla\psi_n|^2$ (blue) along the lines indicated in panel (a), i.e., along the dimer axis for odd states and along the nodal axis for even states. Both experimental and theoretical data are normalized to their respective maximum. The theoretical curves (red and blue) are scaled by the estimated value $k_F = 9.4\,\mathrm{nm}^{-1}$. (c) LDOS at the impurity site $\boldsymbol{r} = \boldsymbol{r}_1$. There are four bound states numbered by 1,2,3,4. Parameters for all theoretical data: $a = 0.6\,k_F$, $\xi_0 = 100/k_F$, $d = 2.5/k_F$, $\eta = 10^{-3}\Delta$, $J = -0.82/N_0$, $U = 0.70/N_0$.

Conductance for STM tips with different orbitals

The electronic current $I_{\mathrm{T}}(\boldsymbol{r}_0,t)$ in the STM tip is given by the rate of change of the particle number operator $N_{\mathrm{T}}(t) = \int_{\mathbb{R}^2} d^2r \sum_\sigma \psi^\dagger_{\mathrm{T}\sigma}(\boldsymbol{r},t)\psi_{\mathrm{T}\sigma}(\boldsymbol{r},t)$ in the tip. Here, $\psi^{(\dagger)}_{\alpha\sigma}(\boldsymbol{r},t)$ denotes the fermionic annihilation (creation) operator of an electron in subsystem $\alpha \in \{\mathrm{T},\mathrm{S}\}$ with spin σ at position $\boldsymbol{r}$ and time t, where T and S denote the STM tip and substrate, respectively. In general, the current signal depends on the position $\boldsymbol{r}_0$ of the STM tip relative to the substrate coordinate system. In general, using the Heisenberg equation of motion, it is defined as

$$I_{\mathrm{T}}(\boldsymbol{r}_0,t) = -e\frac{dN_{\mathrm{T}}(t)}{dt} = -\frac{ie}{\hbar}[V(\boldsymbol{r}_0,t), N_{\mathrm{T}}(t)], \tag{S20}$$

where we define the coupling between the tip and substrate planes by

$$V(\boldsymbol{r}_0,t) = \int_{\mathbb{R}^2} d^2r \int_{\mathbb{R}^2} d^2r' \sum_{\sigma,\sigma'} \Big[\psi^\dagger_{\mathrm{T}\sigma}(\boldsymbol{r},t)V_{\mathrm{T}\sigma,\mathrm{S}\sigma'}(\boldsymbol{r},\boldsymbol{r}')\psi_{\mathrm{S}\sigma'}(\boldsymbol{r}',t) + \psi^\dagger_{\mathrm{S}\sigma'}(\boldsymbol{r}',t)V_{\mathrm{S}\sigma',\mathrm{T}\sigma}(\boldsymbol{r}',\boldsymbol{r})\psi_{\mathrm{T}\sigma}(\boldsymbol{r},t)\Big] \tag{S21}$$

with the hopping $V_{\alpha\sigma,\beta\sigma'}(\boldsymbol{r},\boldsymbol{r}') = V_{\alpha\sigma,\beta\sigma'}\,\delta(\boldsymbol{r}-\boldsymbol{r}'-\boldsymbol{r}_0)$ and matrix elements $V_{\alpha\sigma,\beta\sigma'}$. Using the equal-time fermionic anticommutation $\{\psi_{\alpha,\sigma}(\boldsymbol{r},t), \psi^\dagger_{\beta,\sigma'}(\boldsymbol{r}',t)\} = \delta_{\alpha\beta}\,\delta_{\sigma\sigma'}\,\delta(\boldsymbol{r}-\boldsymbol{r}')$, we arrive at

$$I_{\mathrm{T}}(\boldsymbol{r}_0,t) = \frac{ie}{\hbar}\int_{\mathbb{R}^2} d^2r \int_{\mathbb{R}^2} d^2r' \sum_{\sigma,\sigma'} \Big[V_{\mathrm{T}\sigma,\mathrm{S}\sigma'}(\boldsymbol{r},\boldsymbol{r}')\psi^\dagger_{\mathrm{T}\sigma}(\boldsymbol{r},t)\psi_{\mathrm{S}\sigma'}(\boldsymbol{r}',t) - \psi^\dagger_{\mathrm{S}\sigma'}(\boldsymbol{r}',t)\psi_{\mathrm{T}\sigma}(\boldsymbol{r},t)V_{\mathrm{S}\sigma',\mathrm{T}\sigma}(\boldsymbol{r}',\boldsymbol{r})\Big]. \tag{S22}$$

In order to proceed, let us introduce a bit of notation. First, the dressed lesser Green's function is defined as $G^<_{\alpha\sigma,\beta\sigma'}(\boldsymbol{r},t,\boldsymbol{r}',t') = i\psi^\dagger_{\beta\sigma'}(\boldsymbol{r}',t')\psi_{\alpha\sigma}(\boldsymbol{r},t)$. Second, we define a generalized spacetime convolution between two functions f and g as

$$(f*g)(\boldsymbol{r},t,\boldsymbol{r}',t') = \int_{\mathbb{R}^2} d^2r'' \int_{\mathbb{R}} dt''\, f(\boldsymbol{r},t,\boldsymbol{r}'',t'')g(\boldsymbol{r}'',t'',\boldsymbol{r}',t'). \tag{S23}$$

Third, by extending spin space to include particle-hole degrees of freedom as introduced in Eq. (S1), we define the extended coupling matrices between the tip and substrate as

$$\hat{V}_{\mathrm{ST}}(\boldsymbol{r},t,\boldsymbol{r}',t') = \lambda\,\delta(\boldsymbol{r}-\boldsymbol{r}'-\boldsymbol{r}_0)\,\delta(t-t')\,(\sigma_0 \otimes e^{-i\varphi(t)\tau_3/2}\tau_3), \tag{S24a}$$

$$\hat{V}_{\mathrm{TS}}(\boldsymbol{r},t,\boldsymbol{r}',t') = \lambda\,\delta(\boldsymbol{r}-\boldsymbol{r}'+\boldsymbol{r}_0)\,\delta(t-t')\,(\sigma_0 \otimes e^{i\varphi(t)\tau_3/2}\tau_3). \tag{S24b}$$

Note that $\hat{V}_{\rm ST}(\boldsymbol{r},t,\boldsymbol{r}',t') = \hat{V}^\dagger_{\rm TS}(\boldsymbol{r}',t',\boldsymbol{r},t)$, as required. Here, we introduced $\lambda \geq 0$ that models the coupling strength encoding the spatial distance between the tip and substrate planes. Note also that we introduced the time-dependent phase difference $\varphi(t) = \varphi_0 + \omega_0 t$ between the superconducting tip and the superconducting substrate, where φ_0 is the static phase difference and $\omega_0 = 2eV/\hbar$ is the Josephson frequency that depends on the applied voltage V (see, e.g., Refs. [54, 55] for details). This allows us to write the current as

$$I_{\rm T}(\boldsymbol{r}_0,t) = \frac{e}{2\hbar}\int_{\mathbb{R}^2} d^2r\, {\rm Tr}\Big\{(\sigma_0\otimes\tau_3)\Big[(\hat{V}_{\rm TS}*\hat{G}^<_{\rm ST})(\boldsymbol{r},t,\boldsymbol{r},t) - (\hat{G}^<_{\rm TS}*\hat{V}_{\rm ST})(\boldsymbol{r},t,\boldsymbol{r},t)\Big]\Big\}, \quad \text{(S25)}$$

where the trace runs over spin and particle-hole degrees of freedom and $\hat{G}^<_{\alpha\beta}$ is the lesser Green's function in spin-Nambu space defined by the basis introduced in Eq. (S1).

In general, employing the nonequilibrium Keldysh-Green's function formalism will lead to the appearance of multiple Andreev reflections and the electronic current can be expressed as the Fourier series [54, 55, 58]

$$I_{\rm T}(\boldsymbol{r}_0,t) = \sum_{n=-\infty}^{\infty} I_{{\rm T},n}(\boldsymbol{r}_0)\, e^{in\varphi(t)}. \quad \text{(S26)}$$

To arrive at such a representation, we determine the lesser dressed Green's function $\hat{G}^<_{\alpha\beta}$ by means of the two Dyson equations [59]

$$\hat{G}^<_{\rm ST}(\boldsymbol{r},t,\boldsymbol{r}',t') = \Big[\hat{g}^<_{\rm SS}*\hat{V}_{\rm ST}*\hat{G}^a_{\rm TT} + \hat{g}^r_{\rm SS}*\hat{V}_{\rm ST}*\hat{G}^<_{\rm TT}\Big](\boldsymbol{r},t,\boldsymbol{r}',t'), \quad \text{(S27a)}$$

$$\hat{G}^<_{\rm TS}(\boldsymbol{r},t,\boldsymbol{r}',t') = \Big[\hat{G}^<_{\rm TT}*\hat{V}_{\rm TS}*\hat{g}^a_{\rm SS} + \hat{G}^r_{\rm TT}*\hat{V}_{\rm TS}*\hat{g}^<_{\rm SS}\Big](\boldsymbol{r},t,\boldsymbol{r}',t'). \quad \text{(S27b)}$$

However, since we are only interested in the tunneling limit in which the tip is far away from the substrate, i.e., the tip-to-substrate spacing is large and, hence, the tip-to-substrate coupling $\lambda \ll 1$ is weak, we can replace all local dressed Green's functions $\hat{G}_{\alpha\alpha}$ with bare ones $\hat{g}_{\alpha\alpha}$. In this case, the only non-zero Fourier coefficients in Eq. (S26) are the dc current $I_{{\rm T},0}(\boldsymbol{r}_0)$ and the ac Josephson effect coefficients $I_{{\rm T},\pm 1}(\boldsymbol{r}_0)$.

In the following, we only consider the dc current $I_{{\rm T},0}(\boldsymbol{r}_0)$, which follows from Eq. (S25)

under the assumption $\hat{G}_{\alpha\alpha} \approx \hat{g}_{\alpha\alpha}$ after a lengthy set of manipulations as

$$
\begin{aligned}
I_{\mathrm{T},0}(\boldsymbol{r}_0) = \frac{(2\pi\lambda)^2 e}{2h} \int_{\mathbb{R}^2} d^2r \int_{\mathbb{R}^2} d^2r' \int_{\mathbb{R}} dE \Bigg(\\
&\mathrm{Tr}\Big\{\hat{\rho}_{\mathrm{TT,ee}}(\boldsymbol{r}' - \boldsymbol{r}_0, \boldsymbol{r} - \boldsymbol{r}_0, E - eV)\hat{\rho}_{\mathrm{SS,ee}}(\boldsymbol{r}, \boldsymbol{r}', E)\Big\}[f_{\mathrm{T}}(E - eV) - f_{\mathrm{S}}(E)] \\
&- \mathrm{Tr}\Big\{\hat{\rho}_{\mathrm{TT,hh}}(\boldsymbol{r}' - \boldsymbol{r}_0, \boldsymbol{r} - \boldsymbol{r}_0, E + eV)\hat{\rho}_{\mathrm{SS,hh}}(\boldsymbol{r}, \boldsymbol{r}', E)\Big\}[f_{\mathrm{T}}(E + eV) - f_{\mathrm{S}}(E)]\Bigg).
\end{aligned}
\tag{S28}
$$

Here, $f_\alpha(E) = 1/(1+e^{E/(k_\mathrm{B}T)})$ is the Fermi function of the tip or substrate (assumed to be at the same temperature T with Boltzmann constant k_B). Since the partial trace over particle-hole space has already been performed, the remaining trace runs over the spin degrees of freedom. Furthermore, we defined the matrix spectral function $\hat{\rho}_{\alpha\alpha} = (\hat{g}^a_{\alpha\alpha} - \hat{g}^r_{\alpha\alpha})/(2\pi i)$, which has matrix elements $\hat{\rho}_{\alpha\alpha,\sigma\sigma',\tau\tau'}$ in particle-hole space (index $\tau = \mathrm{e},\mathrm{h}$) and spin space (index $\sigma =\uparrow,\downarrow$). Furthermore, it can be shown that holes result in a factor of 2 in the above equation, which is why the final result simplifies to

$$
I_{\mathrm{T},0}(\boldsymbol{r}_0) = \frac{e}{h} \int_{\mathbb{R}} dE\, \mathcal{T}(E, \boldsymbol{r}_0, V)\, [f_{\mathrm{T}}(E - eV) - f_{\mathrm{S}}(E)], \tag{S29}
$$

where we defined the transmission function

$$
\mathcal{T}(E, \boldsymbol{r}_0, V) = (2\pi\lambda)^2 \int_{\mathbb{R}^2} d^2r \int_{\mathbb{R}^2} d^2r'\, \mathrm{Tr}\Big\{\hat{\rho}_{\mathrm{TT,ee}}(\boldsymbol{r}' - \boldsymbol{r}_0, \boldsymbol{r} - \boldsymbol{r}_0, E - eV)\hat{\rho}_{\mathrm{SS,ee}}(\boldsymbol{r}, \boldsymbol{r}', E)\Big\}. \tag{S30}
$$

This result represents the general dc current between the substrate and the tip as a space-convolution of the two electron-block spin matrices $\hat{\rho}_{\alpha\alpha,\mathrm{ee}}$. It also takes into account the relative position $\boldsymbol{r}_0$ of the tip above the substrate. Note that only the equal-spin diagonal elements of the matrix $\hat{\rho}_{\alpha\alpha,\mathrm{ee}}$ at $\boldsymbol{r} = \boldsymbol{r}'$ can be interpreted as a density of states in agreement with the definition in Eq. (S18).

In the following, we will make a few more simplifying assumptions. We assume that the STM tip has no interesting spin structure, i.e., $\hat{\rho}_{\mathrm{TT,ee}}$ is diagonal and proportional to the identity in spin space, namely $\hat{\rho}_{\mathrm{TT,ee}} = \rho_{\mathrm{TT,ee}}\sigma_0$. Therefore, we are left with $\mathrm{Tr}\{\hat{\rho}_{\mathrm{SS,ee}}\} = \sum_\sigma \rho_{\mathrm{SS},\sigma\sigma,\mathrm{ee}}$.

Wave-function overlap and the derivative rule

Eq. (S30) incoorporates already the overlap integral between tip and substrate wave functions. Let us assume that in subsystem α the orbital described by the wave function $\psi_{\alpha\sigma}(\boldsymbol{r})$ is mainly involved in the tunneling process. Then, $\rho_{\alpha\alpha,\sigma\sigma,\mathrm{ee}}(\boldsymbol{r},\boldsymbol{r}',E) \approx \psi_{\alpha\sigma}(\boldsymbol{r})\psi^*_{\alpha\sigma}(\boldsymbol{r}')\rho_{\alpha\sigma}(E)$, where $\rho_{\alpha\sigma}(E)$ is the energy-dependent density of states. Then, Eq. (S30) simplifies to

$$\mathcal{T}(E,\boldsymbol{r}_0,V) = (2\pi\lambda)^2 \sum_\sigma |M_{\mathrm{ST},\sigma}(\boldsymbol{r}_0)|^2 \rho_{\mathrm{T}}(E-eV)\rho_{\mathrm{S},\sigma}(E), \tag{S31}$$

where the overlap integral matrix elements are

$$M_{\mathrm{ST},\sigma}(\boldsymbol{r}_0) = \int_{\mathbb{R}^2} d^2r\, \psi_{\mathrm{S}\sigma}(\boldsymbol{r})\psi^*_{\mathrm{T}}(\boldsymbol{r}-\boldsymbol{r}_0). \tag{S32}$$

Following Ref. [44], we can assume different orbital wave functions for the STM tip. Then, a tip with an s orbital should result in the tunneling matrix element $M_{\mathrm{ST},\sigma}(\boldsymbol{r}_0) \propto \psi_{\mathrm{S}\sigma}(\boldsymbol{r}_0)$, while a tip with a p_x or p_y orbital should result in $M_{\mathrm{ST},\sigma}(\boldsymbol{r}_0) \propto \kappa^{-1}\partial_x\psi_{\mathrm{S}\sigma}(\boldsymbol{r}_0)$ or $M_{\mathrm{ST},\sigma}(\boldsymbol{r}_0) \propto \kappa^{-1}\partial_y\psi_{\mathrm{S}\sigma}(\boldsymbol{r}_0)$, respectively, which is known as *the derivative rule* [43–45]. Here, κ is the decay constant of the tip wave function. Translating this to our transmission function in Eq. (S30), we choose $\rho_{\mathrm{TT,ee}}(\boldsymbol{r}'-\boldsymbol{r}_0,\boldsymbol{r}-\boldsymbol{r}_0,E-eV) = \delta(\boldsymbol{r}'-\boldsymbol{r}_0)\,\delta(\boldsymbol{r}-\boldsymbol{r}_0)\,\rho_{\mathrm{T}}(E-eV)$ for the s-orbital case to get

$$\mathcal{T}^{(s)}(E,\boldsymbol{r}_0,V) = (2\pi\lambda)^2\rho_{\mathrm{T}}(E-eV)\sum_\sigma \rho_{\mathrm{SS},\sigma\sigma,\mathrm{ee}}(\boldsymbol{r}_0,\boldsymbol{r}_0,E), \tag{S33}$$

which is in agreement with already known results; see, e.g., Ref. [60]. Likewise, we choose $\rho_{\mathrm{TT,ee}}(\boldsymbol{r}'-\boldsymbol{r}_0,\boldsymbol{r}-\boldsymbol{r}_0,E-eV) = \kappa^{-2}\partial_{j'}\delta(\boldsymbol{r}'-\boldsymbol{r}_0)\,\partial_j\delta(\boldsymbol{r}-\boldsymbol{r}_0)\,\rho_{\mathrm{T}}(E-eV)$ for a tip with a p_j orbital ($j = x, y$), which results in

$$\mathcal{T}^{(p_j)}(E,\boldsymbol{r}_0,V) = (2\pi\lambda_p)^2\,\rho_{\mathrm{T}}(E-eV)\sum_\sigma \partial_j\partial_{j'}\rho_{\mathrm{SS},\sigma\sigma,\mathrm{ee}}(\boldsymbol{r},\boldsymbol{r}',E)\Big|_{\boldsymbol{r}=\boldsymbol{r}'=\boldsymbol{r}_0}, \tag{S34}$$

where we absorbed the decay length into an effective new coupling constant $\lambda_p = \lambda/\kappa$ for tunneling through the p orbitals. Since we lack the knowledge about the precise orientation of the orbitals in the x-y-plane, we expect that the only information we get from transport through the p orbitals is about the gradient of the wave function

$$|\nabla\psi(\boldsymbol{r}_0)|^2 = |\partial_x\psi(\boldsymbol{r}_0)|^2 + |\partial_y\psi(\boldsymbol{r}_0)|^2 \propto \Big(\partial_x\partial_{x'}\rho_{\mathrm{SS},\sigma\sigma,\mathrm{ee}}(\boldsymbol{r},\boldsymbol{r}',E) + \partial_y\partial_{y'}\rho_{\mathrm{SS},\sigma\sigma,\mathrm{ee}}(\boldsymbol{r},\boldsymbol{r}',E)\Big)\Big|_{\boldsymbol{r}=\boldsymbol{r}'=\boldsymbol{r}_0}. \tag{S35}$$
